# Clinical significance of the combined test of seven blood coagulation indexes, lipids and platelet agglutination on hypercoagulable state of blood in parturient women

**DOI:** 10.12669/pjms.40.4.7530

**Published:** 2024

**Authors:** Wenjuan Zhang, Tao Yu, Yunshuang Wang, Meili Hu, Tianxiang Gao

**Affiliations:** 1 Wenjuan Zhang, Department of Clinical Laboratory, Baoding Maternal and Child Health Hospital, Baoding 071000, Hebei, China; 2 Tao Yu, Department of Clinical Laboratory, Baoding Maternal and Child Health Hospital, Baoding 071000, Hebei, China; 3 Yunshuang Wang, Department of Clinical Laboratory, Baoding Maternal and Child Health Hospital, Baoding 071000, Hebei, China; 4 Meili Hu, Science and Education Section, Baoding Maternal and Child Health Hospital, Baoding 071000, Hebei, China; 5 Tianxiang Gao, Department of Office, Baoding Municipal Health Supervision Institute, Baoding, 071000, Hebei, China

**Keywords:** Seven blood coagulation indexes, Lipids, Platelet agglutination Test, Parturient women, Hypercoagulable state

## Abstract

**Objective:**

To investigate the value of the combined test of seven blood coagulation indexes, lipids and platelet agglutination on the evaluation of the hypercoagulable state of blood in parturient women.

**Methods:**

This is a retrospective study. Total 50 high risk parturient women who underwent an antenatal examination in Baoding Maternal and Child Health Hospital from June 2021 to January 2023 were selected as the observation group, while 50 normal parturient women who underwent antenatal examination without comorbidities and complications were randomly collected in a ratio of 1:1 as the control group. All subjects had venous blood drawn for testing of seven blood coagulation indexes, lipids and platelet agglutination before delivery, and their general data were recorded.

**Results:**

The activated partial thromboplastin time(APTT) in the observation group was lower than that in the control group, while thrombin time(TT) and D-dimer(DD) were both higher than those in the control group, with statistically significant differences(p<0.05); total cholesterol (TC) and triglyceride (TG) in the observation group were both higher than those in the control group, with statistically significant differences (p<0.05); adenosine diphosphate (ADP) and arachidonic acid (AA) in the observation group were both higher than those in the control group, with statistically significant differences (p<0.05). Moreover, the overall incidence of adverse pregnancy was higher in the observation group than in the control group, with a statistically significant difference (p<0.05).

**Conclusions:**

The combined test of seven blood coagulation indexes, lipids and platelet agglutination results in excellent performance in predicting and judging the presence or absence of the hypercoagulable state of blood in parturient women, with the combination of APTT+TT+DD+TG+ADP+AA being the preferred test.

## INTRODUCTION

During pregnancy, parturient women experience pathophysiological changes in the body, with an increase in coagulation factors and fibrinogen content, leaving the blood system in a hypercoagulable state. At this time, the hypercoagulable state is physiological and the coagulation system is still in dynamic balance, which facilitates hemostasis after delivery.^1^,^2^ However, disruption of the dynamic balance of this hypercoagulable state may predispose a parturient woman to an abnormal hypercoagulable state, which in turn leads to an imbalance in fibrinolytic activity or coagulation-anticoagulation mechanisms. In such a situation, a parturient woman may be at increased risk of thromboembolic disease, postpartum hemorrhage, or disseminated intravascular coagulation (DIC), posing a serious threat to both maternal and fetal health.^3^,^4^

Recent years have witnessed a continued high proportion of high-risk pregnancies and an increased risk of maternal postpartum hemorrhage and thrombotic disorders, with the incidence of postpartum hemorrhage accounting for 2-3% of all deliveries and the incidence of thrombotic disorders in pregnancy ranging from 0.76% to 1.72%.^5^,^6^ The seven blood coagulation indexes and platelet agglutination rate are the main indexes reflecting the coagulation system and fibrinolytic function. A separate test for coagulation indexes is only for a certain stage of the coagulation process but is incapable of a comprehensive, effective and dynamic reflection of the coagulation status. In particular, parturient women during pregnancy have a significant increase in blood volume compared to non-pregnancy, where the volume of plasma increases by 40-50% and exceeds that of red blood cells by 10-15%.^7^

As a result, blood is significantly diluted in normal pregnancy, which also leads to the dilution of blood levels of clotting factors. If a separate test of seven blood coagulation indexes and platelet agglutination is conducted at this time, only the plasma levels of coagulation factors and platelet function at a certain stage will be tested, which is non-dynamic and leads to poor accuracy of the results. Changes in the internal environment during pregnancy also include disturbances in lipid metabolism, mainly in the form of elevated lipid levels. As a consequence, hyperlipidemia can occur and correspondingly significantly increase the occurrence of secondary blood hypercoagulability in parturient women.^8^

Theoretically, prenatal monitoring of coagulation index, blood lipid level and platelet agglutination function can all play an auxiliary role in judging the coagulation state, which is of great significance for preventing related complications in parturient women. In this study, a retrospective analysis was conducted on the clinical data of women who underwent antenatal examinations in the Obstetrics and Gynecology Clinic of Baoding Maternal and Child Health Hospital, in order to investigate the clinical significance of the combined test of seven blood coagulation indexes, lipids and platelet agglutination in judging maternal hypercoagulable state.

## METHODS

This is a retrospective study. Patient data including demographic data were retrieved from electronic medical record systems. Fifty high-risk parturient women who underwent an antenatal examination in Baoding Maternal and Child Health Hospital from June 2021 to January 2023 were selected as the observation group, while 50 normal parturient women who underwent antenatal examination without comorbidities and complications in Baoding Maternal and Child Health Hospital were randomly collected in a ratio of 1:1 as the control group.

### Ethical Approval

The study was approved by the Institutional Ethics Committee of Baoding Maternal and Child Health Hospital (No.:2023-01-K007; date: May 12, 2023), and written informed consent was obtained from all participants.

### Inclusion criteria:

- Parturient women aged 20-45 years.
- Parturient women confirmed by color ultrasound as intrauterine pregnancy.
- Parturient women who neither have coagulation disorders nor have taken medications that affect coagulation.
- Parturient women and their family who agreed to this study and signed the informed consent form.

### Exclusion criteria:

- Patients with oral anticoagulant drugs during pregnancy.
- Patients with gestational hypertension, gestational diabetes, etc.
- Patients with severe abnormal liver and kidney function.
- Patients with hematological diseases or diseases affecting coagulation function (skin lesions, submucosal bleeding, gingival bleeding, gastrointestinal or other visceral bleeding).
- Patients with tumors and other diseases

### Specimen collection and processing

Fasting venous blood 6ml was collected from parturient women in the early morning, of which 2ml was sent for testing of seven blood coagulation indexes, 2ml for testing of blood lipids, and two ml for testing of platelet agglutination. Within two hours after blood collection, the specimens were tested and analyzed by dedicated personnel according to the operating rules.

### Observation indexes

Seven blood coagulation indexes, including activated partial thromboplastin time (APTT), plasma prothrombin time (PT), thrombin time (TT), fibrinogen (FIB), fibrinogen degradation product (FDP), D-dimer (DD), and antithrombin-III (AT-III) were analyzed by STA-R Evolution Automatic Blood Clots Instrument manufactured by Stago, a French company. The lipid levels were analyzed by Hitachi 7180 Automatic Biochemical Analyzer, and the observed items included total cholesterol (TC), triglyceride (TG), high-density lipoprotein (HDL), low-density lipoprotein (LDL) and very low-density lipoprotein (VLDL). The platelet agglutination rate was analyzed by the AG400 Semi-automatic Platelet Aggregometer manufactured by Shandong Telesyn Medical Technology Co., Ltd. using adenosine diphosphate (ADP) and arachidonic acid (AA) as the inducing agents.

### Statistical analysis

All data in this study were statistically analyzed by SPSS 20.0 software. The measurement data were expressed as () using two independent samples *t* test. Count data were expressed as n (%), which were compared between groups using χ^2^ test. The ROC curve was used to determine the diagnostic value with p<0.05 indicating a statistically significant difference.

## RESULTS

The age, gestational age, body mass index and cesarean section rate in the observation group were higher than those in the control group, with statistically significant differences (p<0.05), but there was no statistically significant difference in the number of pregnancies between the two groups (p>0.05), Table-I.

**Table-I:** Comparison of general data between the two groups (n=50).

Group	Age (years old)	Gestational age (weeks)	BMI (kg/m^2^)	Number of pregnancies		Delivery method	

				Multiparity	Primiparity	Normal delivery	Cesarean delivery
Observation group	34.62±6.20	36.20±2.67	37.76±2.79	11	39	26	39
Control group	32.20±5.25	34.76±3.12	36.26±3.36	15	35	24	11
t/χ^2^ value	2.105	2.478	2.434	0.832		7.429	
*P* value	0.038	0.015	0.017	0.362		0.006	

Regarding the seven blood coagulation indexes, APTT in the observation group was lower than that in the control group, while TT and DD were both higher than that in the control group, with statistically significant differences (p<0.05), while the differences in the remaining blood coagulation indexes were not statistically significant between the two groups (p>0.05). In terms of lipid indexes, TC and TG in the observation group were higher than those in the control group, with statistically significant differences (p<0.05), while there was no statistically significant difference in other lipid indexes (p>0.05). Both ADP and AA in the observation group were higher than those in the control group, with statistically significant differences (p<0.05), Table-II.

**Table-II:** Comparison of seven blood coagulation indexes, lipids and platelet agglutination between the two groups (*χ̅*±*S*).

Item	Observation group	Control group	t value	P value
*Seven blood coagulation indexes*				
APTT(sec)	30.15±4.92	32.90±5.36	2.668	0.009
PT(sec)	12.43±4.17	12.36±4.18	0.091	0.928
TT(sec)	16.57±3.90	14.87±4.10	2.123	0.036
FIB(g/L)	5.32±2.23	5.19±2.20	0.280	0.780
FDP(mg/L)	6.46±3.74	6.34±4.07	0.148	0.882
DD(mg/L)	2.42±0.36	1.86±0.31	8.339	0.000
AT-III(g/L)	115.19±13.51	113.73±10.59	0.600	0.550
Lipids				
TC(*mmol*/L)	9.33±2.16	8.54±1.62	2.057	0.042
TG(*mmol*/L)	2.55±0.45	2.15±0.40	4.676	0.000
HDL(*mmol*/L)	1.09±0.24	1.04±0.24	1.043	0.299
LDL(*mmol*/L)	3.68±0.42	3.55±0.39	1.529	0.129
VLDL(*mmol*/L)	1.15±0.31	1.05±0.34	1.542	0.126
*Platelet agglutination*				
*ADP*(%)	66.18±6.71	63.52±5.23	2.212	0.029
AA(%)	75.76±3.75	72.68±6.70	2.837	0.006

The overall incidence of various indexes such as venous thromboembolism, (premature rupture of membranes (PROM), DIC, postpartum massive hemorrhage, neonatal death and maternal death in the observation group was higher than that in the control group, with a statistically significant difference (p<0.05), Table-III.

**Table-III:** Comparison of pregnancy outcomes between the two groups [*n*, (%)].

Group	n	Thromboembolism	PROM	DIC	Postpartum massive hemorrhage	Neonatal death	Maternal death	Total
Observation group	50	3(6.00)	5(10.00)	2(4.00)	2(4.00)	1(2.00)	1(2.00)	14(28.00)
Control group	50	2(4.00)	1(2.00)	1(2.00)	1(2.00)	0	0	5(10.00)
*χ^2^* value								5.263
*P* value								0.022

**Fig.1:**
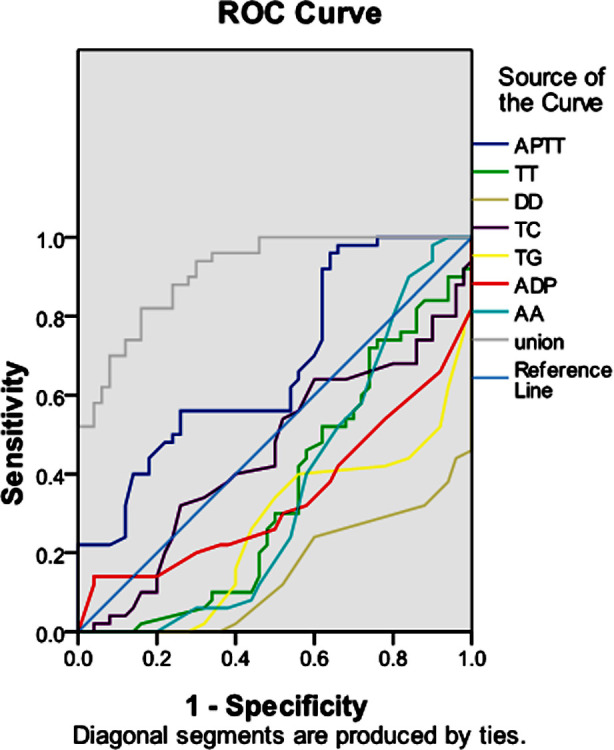
Prediction of a hypercoagulable state by ROC curve.

The ROC curve plotted by combining the seven indexes APTT, TT, DD, TC, TG, ADP, and AA with lipids and platelet agglutination in both groups showed that the combined test was of predictive value for the hypercoagulable state of blood in parturient women, with the area under the ROC curve being 0.914, which was significantly higher than that of the single screening test, with a statistically significant difference (p<0.05), Table-IV and Fig.1.

**Table-IV:** Value of seven blood coagulation indexes, lipids and platelet agglutination tests in predicting hypercoagulation status of parturient women.

	APTT	TT	DD	TC	TG	ADP	AA	Combination
AUC	0.675	0.352	0.149	0.454	0.264	0.342	0.355	0.914
95%CI	0.569-0.780	0.243-0.460	0.076-0.221	0.340-0.568	0.167-0.361	0.233-0.451	0.243-0.467	0.862-0.966
*P* value	0.003	0.011	0.000	0.426	0.000	0.006	0.012	0.000

## DISCUSSION

In the present study, APTT in parturient women in the observation group was significantly lower than in normal pregnancy, while TT and DD were significantly higher than in normal pregnancy, carrying statistically significant differences (p<0.05), which is consistent with the literature. In a normal organism, coagulation and anticoagulation are in dynamic balance, whereas in pregnancy, the coagulation and fibrinolytic functions of the organism are physiologically altered. Especially in late pregnancy, several coagulation factors, platelet factor IV and fibrinogen increase, while antithrombin levels decrease.

This is complemented by other hypercoagulable factors such as increased blood lipids, increased prothrombin substances, as well as increased pelvic venous pressure, low activity and slowed venous blood flow during late pregnancy, resulting in a hypercoagulable state of the body during pregnancy, which is particularly pronounced during late pregnancy.^9^,^10^ A hypercoagulable state is mainly defined as a decrease in the body’s anticoagulant function and an increase in coagulation. During pregnancy, moderate hypercoagulation is a physiological protective measure for the mother, allowing maternal fibrin deposition between the arteries, placental villi and uterine wall.

This maintains the integrity of the placenta, the formation of thrombus during placental abruption, and serves to stop bleeding rapidly during labor and after delivery. Thus, hypercoagulation is an important mechanism to prevent postpartum hemorrhage.^11^ At the same time, secondary fibrinolytic activity is activated during coagulation, allowing rapid clearance of thrombus in the uterine venous sinuses and spiral arteries and accelerating the regeneration and repair of the endometrium after delivery.^12^ It was shown in relevant studies^13^,^14^ that during normal pregnancy, most anticoagulant substances decrease and coagulation factors increase, with a gradual decrease in APTT and PT and a gradual increase in TT, FDP and DD as the gestational week increases.

During pregnancy, the coagulation-anticoagulation and fibrinolytic-antifibrinolytic systems are altered, leaving the coagulation system of parturient women in a hypercoagulable state. This hypercoagulable state is in a dynamic equilibrium, which under normal circumstances is a physiological protection for parturient women, under the stimulation of pathological factors. Once this dynamic equilibrium is disrupted, parturient women, especially those at high risk, are prone to various coagulation abnormalities, leading to the emergence of an abnormal hypercoagulable state and an imbalance in the coagulation-anticoagulation mechanism, leading to multiple complications in pregnancy and serious threats to maternal and fetal safety.^15^

It was shown by Erez O et al. and Mannaerts D et al.^16^,^17^ that parturient women with gestational hypertension, diabetes mellitus, eclampsia and advanced age and overweight have a significantly increased risk of adverse outcomes such as venous thromboembolic disease, postpartum hemorrhage and DIC compared to normal parturient women, indicating that hypercoagulable state significantly affects maternal healing and seriously endangers the health and life safety of mother and child. In this study, age and BMI in the observation group were significantly higher than those in the normal control group, with statistically significant differences (p<0.05); In the comparison of adverse events in the prodromal outcome between the two groups, the overall incidence of adverse events in the observation group was higher than that in the control group, with a statistically significant difference (p<0.05). This is consistent with the clinically reported results.

Antza C et al.^18^ showed that blood volume increases by about 40% at the end of pregnancy compared to non-pregnancy, and the increase in blood volume is most pronounced in plasma, which significantly exceeds the increase in the number of red blood cells. As a result, blood is diluted in normal pregnancy, which leads to a dilution of the clotting factor content in the blood as well. Changes in maternal lipid levels have a close bearing on changes in physiological and biochemical factors in the body during pregnancy. Besides, advanced age and obesity also contribute to higher than normal lipid levels. Specifically, elevated lipid levels lead to the development of hyperlipidemia, which significantly increases the occurrence of secondary coronary vascular disease in pregnant women, while pregnancy has an impact on lipid levels and lipoprotein metabolism.

It was shown in Lewicka I et al.^19^ that the formation of a hypercoagulable state in parturient women is closely related to their high blood lipids. Platelet agglutination is a vital physiological property of platelets and is an important factor in their involvement in hemostasis and thrombosis. The platelet agglutination test is an important clinical method for the diagnosis of prethrombotic state and thrombotic disorders and is currently considered the gold standard ADP and AA-induced photopigmentation method.^20^ In this study, the age, BMI, gestational week and mode of delivery of parturient women in the observation group were significantly different from those in the control group. It was believed that the older age, higher BMI and longer gestational week of the parturient women in the observation group led to their hypercoagulable blood and more complications during delivery, and some of them underwent cesarean delivery for rapid delivery and to reduce the occurrence of comorbidities.

### Limitations

Nevertheless, the small sample size caused limitations in this study and affected the level of evidence to some extent. To address this, more sample sizes will be included in future studies to obtain more accurate clinical results.

## CONCLUSION

The combined test of seven blood coagulation indexes, lipids and platelet agglutination boast excellent performance in predicting and judging the presence or absence of the hypercoagulable state of blood in parturient women, with the combination of APTT+TT+DD+TG+ADP+AA being the preferred test. It is of important clinical significance for the prevention of thrombosis, pre-thrombotic states and complications associated with hypercoagulable states in pregnant women.

### Authors’ Contributions:

**WZ** and **TY:** Carried out the studies, participated in collecting data, drafted the manuscript, are responsible and accountable for the accuracy and integrity of the work.

**YW:** Performed the statistical analysis and participated in its design.

**MH** and **TG:** Participated in acquisition, analysis, or interpretation of data and draft the manuscript.

All authors read and approved the final manuscript.

## References

[1] Tanaka KA, Bharadwaj S, Hasan S, Judd M, Abuelkasem E, Henderson RA (2019). Elevated fibrinogen, von Willebrand factor, and Factor VIII confer resistance to dilutional coagulopathy and activated protein C in normal pregnant women. Br J Anaesth.

[2] Naime ACA, Ganaes JOF, Lopes-Pires ME (2018). Sepsis:The Involvement of Platelets and the Current Treatments. Curr Mol Pharmacol.

[3] Yao H, Ji Y, Zhou Y (2022). Analysis of blood coagulation indexes, thromboelastogram and autoantibodies in patients with recurrent pregnancy loss. Pak J Med Sci.

[4] Chen Y, Lin L (2017). Potential Value of Coagulation Parameters for Suggesting Preeclampsia During the Third Trimester of Pregnancy. Am J Med Sci.

[5] Scheres LJJ, Lijfering WM, Groenewegen NFM, Koole S, de Groot CJM, Middeldorp S (2020). Hypertensive Complications of Pregnancy and Risk of Venous Thromboembolism. Hypertension.

[6] Danesh J, Lewington S, Thompson SG, Lowe GD, Collins R, Fibrinogen Studies Collaboration (2005). Plasma fibrinogen level and the risk of major cardiovascular diseases and nonvascular mortality: an individual participant meta-analysis. [published correction appears in JAMA. 2005;294(22):2848. JAMA.

[7] Duygu H, Turkoglu C, Kirilmaz B, Turk U (2008). Effect of mean platelet volume on postintervention coronary blood flow in patients with chronic stable angina pectoris. J Invasive Cardiol.

[8] Reese JA, Peck JD, Deschamps DR, McIntosh JJ, Knudtson EJ, Terrell DR (2018). Platelet Counts during Pregnancy. N Engl J Med.

[9] Croles FN, Nasserinejad K, Duvekot JJ, Kruip MJ, Meijer K, Leebeek FW (2017). Pregnancy, thrombophilia, and the risk of a first venous thrombosis:systematic review and bayesian meta-analysis. BMJ.

[10] Tomimatsu T, Mimura K, Matsuzaki S, Endo M, Kumasawa K, Kimura T (2019). Preeclampsia:Maternal Systemic Vascular Disorder Caused by Generalized Endothelial Dysfunction Due to Placental Antiangiogenic Factors. Int J Mol Sci.

[11] Reese JA, Peck JD, Deschamps DR, McIntosh JJ, Knudtson EJ, Terrell DR (2018). Platelet Counts during Pregnancy. N Engl J Med.

[12] Jacobson B, Rambiritch V, Paek D, Sayre T, Naidoo P, Shan J (2020). Safety and Efficacy of Enoxaparin in Pregnancy:A Systematic Review and Meta-Analysis. Adv Ther.

[13] Scheres LJJ, Lijfering WM, Groenewegen NFM, Koole S, de Groot CJM, Middeldorp S (2020). Hypertensive Complications of Pregnancy and Risk of Venous Thromboembolism. Hypertension.

[14] Adachi T (2019). Pregnancy and labor management:women with venous thromboembolism or associated significant risk factors. Rinsho Ketsueki.

[15] Bagot CN, Leishman E, Onyiaodike CC, Jordan F, Gibson VB, Freeman DJ (2019). Changes in laboratory markers of thrombotic risk early in the first trimester of pregnancy may be linked to an increase in estradiol and progesterone. Thromb Res.

[16] Erez O (2017). Disseminated intravascular coagulation in pregnancy - Clinical phenotypes and diagnostic scores. Thromb Res.

[17] Mannaerts D, Heyvaert S, De Cordt C, Macken C, Loos C, Jacquemyn Y (2019). Are neutrophil/lymphocyte ratio (NLR), platelet/lymphocyte ratio (PLR), and/or mean platelet volume (MPV) clinically useful as predictive parameters for preeclampsia?. J Matern Fetal Neonatal Med.

[18] Antza C, Cifkova R, Kotsis V (2018). Hypertensive complications of pregnancy:A clinical overview. Metabolism.

[19] Lewicka I, Kocyłowski R, Grzesiak M, Gaj Z, Sajnóg A, Barałkiewicz D (2019). Relationship between pre-pregnancy body mass index and mineral concentrations in serum and amniotic fluid in pregnant women during labor. J Trace Elem Med Biol.

[20] Chasan-Taber L, Silveira M, Waring ME, Pekow P, Braun B, Manson JE (2016). Gestational Weight Gain, Body Mass Index, and Risk of Hypertensive Disorders of Pregnancy in a Predominantly Puerto Rican Population. Matern Child Health J.

